# Visual encoding of social cues predicts sociomoral reasoning

**DOI:** 10.1371/journal.pone.0201099

**Published:** 2018-07-25

**Authors:** Mathieu Garon, Marie Maxime Lavallée, Evelyn Vera Estay, Miriam H. Beauchamp

**Affiliations:** 1 Department of Psychology, University of Montreal, Montreal, Qc, Canada; 2 Centro de Desarrollo de Tecnologías de Inclusión, Escuela de Psicología, Pontificia Universidad Católica de Chile, Macul, Chile; 3 Sainte-Justine Hospital Research Center, Montreal, Qc, Canada; Cardiff University, UNITED KINGDOM

## Abstract

As the first step of social information processing, visual encoding underlies the interpretation of social cues. Faces, in particular, convey a large amount of affective information, which can be subsequently used in the planning and production of adaptive social behaviors. Sociomoral reasoning is a specific social skill that is associated with engagement in appropriate social behaviors when faced with dilemmas. Previous studies using eye tracking suggest that visual encoding may play an important role in decision-making when individuals are faced with extreme moral dilemmas, but it is not known if this is generalizable to everyday situations. The main objective of this study was to assess the contribution of visual encoding to everyday sociomoral reasoning using eye tracking and ecological visual dilemmas. Participants completed the SocioMoral Reasoning Aptitude Level (SoMoral) task while their eye movements and pupil dilation were recorded. While visual encoding was not a predictor of sociomoral decision-making, sociomoral maturity was predicted by *fixation count*. Thus, in an ecological context, visual encoding of social cues appears to be associated with sociomoral maturity: the production of a justification is associated with volitional encoding strategies. Implications with regards to the dual-process theory of sociomoral reasoning and social information processing are discussed.

## Introduction

Sociomoral reasoning is an essential process in social interactions and influences decisions made in everyday life from a very young age [1,2]. It can involve elaborate reasoning processes, while also relying on rapid and automatic cognitive bases. In most situations, production of a moral behavior involves intuitive processes such as perceptual encoding of the situation, which is required to extract relevant information from the context [3,4]. As a significant first step in the process, visual encoding is likely to play an important role in many subsequent aspects of sociomoral reasoning. Observing eye movements is a reliable way to assess visual encoding in social situations and studies in the fields of social and affective neurosciences increasingly rely on eye tracking techniques to effectively assess the relation between visual encoding and social information processing or social cognition. A limited number of existing studies have explored the association between visual encoding and moral decision-making and their findings are contradictory. Moreover, the methods used previously do not always allow generalization of the results to everyday social contexts. The current study aimed to assess the role of visual encoding of social information in sociomoral reasoning using a task presenting everyday dilemmas and eye-tracking. Understanding the link between visual encoding and sociomoral reasoning has multiple implications on our grasp of the cognitive processes at play in social interactions and their alteration over the course of development and in clinical conditions.

### Moral reasoning

Moral reasoning is a complex cognitive process used to make a decision, to produce a judgment, and to justify it in the context of a moral dilemma. Sociomoral reasoning refers to the type of moral reasoning that takes place in a social context and that places emphasis on social interaction to define what is morally right [5]. Given the dearth of literature in the area of visual encoding and moral processes, both the classical and social aspects of moral reasoning are considered here. Of note, moral decision-making refers to the finality of the moral reasoning process, that is, the choice of an action (which generally excludes its justification), while the term “moral maturity” refers to the quality of the justification provided by an individual to support a choice or action, and can be quantified as consecutive levels [6]. For example, an individual who decides not to steal (decision-making) could justify their choice by mentioning the fear of being punished (low level of moral maturity) or by the universal principle of right to property (high level of moral maturity).

Moral reasoning has been defined by some as an explicit and deliberative process. Foundational theories of cognitive development [7,8] considered that the ability to reason about moral dilemmas is an acquired skill like reading or problem solving, suggesting that children gradually develop their potential to process environmental information to produce morally acceptable behaviors [9]. According to Kohlberg [10], deliberative reasoning processes are the basis of morality and the development or moral reasoning occurs across several stages that mirror the maturation of reasoning skills and cognition more generally. As such, the quality of moral reasoning can be evaluated on the basis of the justification produced to support a decision. Consistent with the idea of a mainly deliberative moral reasoning, predictors of moral reasoning and decision-making include cognitive functions that are involved in deliberation in general, such as executive functions [11], intellectual functions [12], general reasoning abilities [13], and performance on logical reasoning tasks [14].

On the other hand, the involvement of implicit, automatic, and intuitive processes in moral reasoning has also been documented [3,15,16]. The social intuitionist thesis [3] purports that when individuals are faced with a moral dilemma, their responses are generated quickly and effortlessly without having to explicitly formulate the dilemma. When asked to justify their decision, individuals are thought to produce and provide judgements and justification arguments *a posteriori*. The justification elicited is therefore not necessarily linked with the cognitive process underlying decision-making, but serves socially adaptive functions such as defending the decision to others, which helps to reduce any cognitive dissonance associated with decision-making. The phenomenon of *moral dumbfounding* [17], which occurs when individuals respond quickly to a dilemma and are then unable to justify their decision [18], supports the intuitionism thesis. The fact that a decision can be made without explicit volition suggests that it is possible (without always being the case) to produce a moral judgment completely intuitively. Consistent with this, emotion and affective state are often described as a significant and intuitive part of moral reasoning and have been shown to consistently influence moral decision-making [19–21]. Activation of the sympathetic nervous system is an implicit aspect of the affective experience that is associated with moral reasoning. This has been documented using skin conductance [22–24] and the findings indicate that production of a moral decision is generally associated with higher sympathetic activation when compared to an immoral decision. In sum, while it is still unclear if moral reasoning can be defined as completely driven by intuitive processes, they certainly play a role in the outcome.

Efforts have been made to integrate the intuitive and deliberative views of moral reasoning and there is a growing consensus that both aspects are important [16,25–27]. According to this dual-system conception, moral reasoning is influenced by emotions or by reason depending on the characteristics of the dilemma. Studies of moral decision-making using adaptations of the *Trolley Problem* offer some insight into the subject [28–30]. This scenario, extensively used in moral reasoning research [31–33], is about a runaway trolley headed toward five railway workers. Participants are informed that they have the possibility to flip a switch that will lead the trolley down a side track on which another person is working. Essentially, in this type of dilemma, participants are asked to choose between a utilitarian decision (leading to the greatest good for the greatest amount of people) and a deontological decision (based on immutable universal moral principles). Variations of this problem are associated with a greater proportion of either utilitarian or deontological responses. Differences between participants’ affective response may account for some of this discrepancy: problems that elicit a greater affective response lead participants to rely more on intuition, which is associated with deontological decisions, whereas deliberation is associated with utilitarian judgments [16]. In sum, it is generally recognized that, while moral reasoning is a deliberative, goal-oriented, process, it can also involve intuitive processes. The relative weight of these two aspects is likely to be modulated by characteristics of the task.

### Moral reasoning assessment

Moral reasoning is typically studied using questionnaires composed of vignettes exposing conflictual situations (moral dilemmas) in which participants must choose one outcome and justify it [34] or using moral judgment tasks in which participants judge the morality of other peoples’ actions [15]. One of the main criticisms of traditional moral reasoning paradigms is that they suffer from limited ecological validity [35–37]. The vignettes are usually presented in the form of short texts or cartoon illustrations. This presentation is not representative of real life moral conflicts because it decreases the amount of peripheral and contextual information available to participants and because it is typically from a third-person point-of-view. It may also introduce bias in the reasoning of participants by providing a structure to the moral dilemma that they would have to infer otherwise [38]. Because of this, traditional moral dilemmas are unlikely to elicit the same emotional reactions in participants as real-life events. Moreover, classic moral dilemmas, such as the trolley problem, typically present situations that are extreme (e.g. of life and death) and have little chance of actually occurring. They are thus not closely linked with everyday social behaviors. In addition, some paradigms are problematic because of the cognitive confounds they introduce. For example, understanding the moral issues in the Defining Issues Test [39] and the Socio-Moral Reflection measure [40] is dependent on advanced reading skills [41]. Finally, measuring moral maturity (the quality of the justification produced) after the dilemma has been presented, while interesting, presents limitations inherent to self-report measures: it is not possible to assume that the justification is a valid account of the reasoning steps leading to moral decision-making. Instead, moral maturity level ideally should be associated with objective measures of reasoning that contribute decision-making.

The *Socio-Moral Reasoning Ability Level* task (SoMoral) [1,11,42–44] addresses some of these methodological limitations. The task consists of visual dilemmas that represent moral conflicts (e.g. concerns with justice, welfare/harm and rights) according to Social Domain Theory [45]. This theory defines morality, based on fairness, justice, welfare and rights, as one of the three main forms of social knowledge (moral, societal and psychological) that guide social interactions and decisions. The pictures of the SoMoral are presented in first-person perspective, contain no written text, and are representative of social situations in daily life and can therefore be considered as sociomoral conflicts rather than purely moral ones. The task nevertheless maintains the main features of classic moral dilemmas. For example, participants are asked to provide a decision and justify it. The type of response and the quality of the justification are assessed using a 5-level cognitive-developmental scoring system. The design of the SoMoral lends itself well to the use of eye tracking without having to change the paradigm, thus presenting the possibility of ecological and non-invasive measurement of visual information processing during moral reasoning.

### Moral reasoning and visual encoding

The relevance of studying the earliest stages of information processing in social cognition and, more broadly, in the production of appropriate social behaviors has been noted [46,47]. As moral reasoning shares core characteristics with other social cognitive processes [48], information processing in the context of moral dilemmas is similar to information processing in any social situation. The *Social Information Processing model* [49] proposes six steps: Encoding, Interpretation, Clarification of the objectives, Responses development, Choice of a behavior and Action. The first step—encoding of social cues—involves the detection of relevant cues in the environment to understand a situation and therefore has an essential role in social reasoning and the production of adaptive behaviors: if the information is not encoded properly, all subsequent steps are likely to be affected. An individual who does not adequately perceive social cues could develop a biased understanding of a given social situation and could consequently be at risk for engaging in maladaptive behaviors. As proposed in the dual-process theory of moral reasoning, the Social Information Processing model suggests that the production of an adaptive social behavior relies on both intuitive and deliberative processes, visual encoding being one of the former.

People generally present an encoding bias to social cues such as faces because they contain a large amount of information on context. It is therefore not surprising that this bias constitutes an adaptive advantage and that it is a critical basis of social functioning [4,50]. Faces in particular are considered to be a potent and rich source of information that can shape social behaviors [51]. Many characteristics of the actors in a scene can be established quickly and effortlessly from faces alone [52]. Inferred characteristics subsequently influence behavior toward individuals [53] and day-to-day decisions [54,55]. In addition, face processing can also inform about important situational elements (e.g. intentions of others, presence of a threat). Although the precise cognitive mechanisms underlying the link between face processing and prosocial behaviors are still largely unknown, it is logical to assume that they may also be involved in sociomoral reasoning. Individuals who pay greater attention to social cues when faced with a sociomoral dilemma are more likely to gather the information needed to make a moral choice.

Eye tracking is modern, cost-effective, and non-invasive way of measuring visual fixation patterns to social stimuli in real-time, and therefore provides a way of quantifying intuitive encoding processes by offering insight into the strategies and biases used in social information possessing, decision-making and moral reasoning. The method has significant advantages in the study of decision-making and moral reasoning compared to self-report measures [56,57]. To date, the studies that have explored moral reasoning in conjunction with eye tracking have adapted classic moral paradigms to do so. For example, in two studies [58,59], moral dilemmas were presented to participants in the form of short texts. Two pictures were presented after the text, each corresponding to one of the response options. Both studies led to similar results suggesting that visual encoding is biased toward the image that represents their response choice. Kastner [58] found that a longer visual fixation time was allocated to pictures of people that the participants decided to save when compared to those that they decided to sacrifice. Similarly, Pärnamets [59] found that longer fixation times are allocated to the chosen option. In the same vein, later work by Pärnamets and colleagues [60] indicates that moral judgment can be influenced using eye tracking by manipulating the timing of the question. That is, when participants have to provide an answer to a moral dilemma at the same time as their gaze is on one of the options, the likelihood that the participant choses this option increases. Conversely, a study using a similar paradigm in virtual reality [61] reports the opposite pattern of results, with participants allocating more fixation time to victims in moral dilemmas. Similarly, Decety and colleagues [15] measured the eye movements of participants who were asked to evaluate the morality of others’ actions and observed a longer fixation time on the victims of wrongdoing. These studies do not offer a clear pattern of findings, which might stem from discrepancies in the methodologies used (e.g., use of real-time dynamic virtual reality *versus* static text reading; first *versus* third person perspectives).

Pupillometry is a reliable indicator of autonomic arousal response to affective stimuli that can be used in the context of eye tracking and can provide supplemental information on the intuitive processes involved in decision-making, more generally, and moral reasoning [62]. For instance, studies in decision-making in general typically found an increase in pupil dilation 2000 ms prior to decision-making [59,63]. In their study, Skulmowski and colleagues [61] found that an increase in pupil size occurs following decision-making, which may be associated with the post-decisional consolidation of the selected outcome. However, neither of these studies specifically linked arousal (pupil dilation) and moral reasoning (decision-making or justification).

The eye tracking studies reviewed here allow a more objective, real-time, assessment of perceptual processes involved in moral reasoning. By using visual stimuli, they are also a significant step towards a more ecological approach to the study of moral reasoning, in comparison with conventional paper-pencil measures. They nonetheless present methodological challenges associated with the format, timing and interpretation of the moral reasoning paradigms traditionally used. It is also difficult to generalize the results of extreme moral dilemmas to everyday life. It is likely that visual encoding of social cues is also important for everyday sociomoral reasoning, but this has not been studied.

### Aims and hypotheses

This study investigates the role of visual information encoding in sociomoral reasoning using an ecological task and eye tracking techniques. The aim was to determine whether eye movements are predictive of sociomoral reasoning. We expected that visual encoding of social information would be significantly associated with the number of socially adaptive decisions (sociomoral decision-making) and with the level of sociomoral justifications produced (sociomoral maturity) on the SoMoral task. Specifically, we hypothesized that the total number of fixation (*fixation count*) on social cues would be positively associated with the number of morally adaptive decisions and with greater sociomoral maturity. We further expected that the number of fixation produced between the onset of social cues and the first fixation toward them (*fixations before*) would be negatively associated with sociomoral decision-making and sociomoral maturity. Finally, we hypothesized that pupillary response (pupil dilation), as an indicator of autonomic arousal associated with affective response, would be positively associated with sociomoral decision-making, but not necessarily with sociomoral maturity, since justification is likely the result of a deliberative process that may be post-decisional and not directly linked with the affective response.

## Materials and methods

### Design

This study was a confirmatory correlational study. Scores on a sociomoral reasoning task (sociomoral decision-making and sociomoral maturity score) were the dependent variables. Eye movements (*fixations before*, *fixation count*) and pupillary dilation were the independent variables.

### Participants

All participants provided informed written consent for the study. The study was approved by the University of Montreal Research Ethics Committee (Comité d'éthique de la recherche en arts et en sciences, CERAS). The approval number is CÉRFAS-2011-12-016-P. Sixty participants (26 women, 43.3%) aged 16 to 44 years old (M = 23.02, SD = 6.56 years) completed the study. The inclusion criteria were i) aged between 14 and 50 years; (ii) fluent in French or English, and (iii) no documented psychological, developmental or neurological disorder. All participants were francophone, were blind to the study aim and hypotheses. They were recruited in the community, but the majority were college and university students. They received a monetary compensation of twenty dollars for their time and parking fees.

### Measures

#### Sociomoral reasoning

Participants completed the Socio-Moral Reasoning Aptitude Level task [42]. The SoMoral was initially designed for use with adolescent participants, but is also validated with young adults [1]. It is a self-paced, visual, computer-based task with gender-appropriate versions. In this study, the 16 dilemmas version was used. Each dilemma consists of an introductory screen presenting the name of the dilemma, three first-person perspective pictures of actors playing out various social scenarios representing a sociomoral conflict and a final screen presenting a dichotomous decision (whether or not to engage in a particular action). Examples of items from the task have been presented in previous articles [1,11]. The introductory screen presents the name of the dilemma. A fixation cross is then presented in the middle of the screen for one second. A social situation involving a sociomoral dilemma is then presented via three first-person perspective pictures (e.g., A woman is walking and her wallet is about to fall out of her handbag (picture 1); the wallet falls out on the sidewalk while the woman continues on her way (picture 2); the participant finds the wallet and his/her friends are happy to see the money suggesting they should keep it (picture 3). The following screen presents a dichotomous decision choice and in the final screen participants are then asked to provide a justification for their decision, which is recorded verbatim for subsequent coding. The dilemmas include situations likely to occur in everyday life (e.g. a classmate asks for the answers during an exam and the participant must decide whether or not to give their answer; the participant has the opportunity to cheat while playing a board game and is asked if they would or not; the participant accidentally breaks the windshield of a car window and is asked if they would tell the owner of the car or not). Each picture was presented for three seconds and each scenario was preceded by a fixation cross in the middle of the screen. The aggregate number of socially adaptive responses to the dichotomous decision question was compiled to obtain a sociomoral decision-making score, which ranges from 0 to 16 points. Participants were then asked to provide a justification for their decision. Each participant’s justification was recorded verbatim and scored using a standardized coding system [43] based on an adaptation of the cognitive-developmental approach to sociomoral reasoning [6]. Developmental stages of sociomoral reasoning have been updated and adapted to fit the social nature of the dilemmas in the SoMoral task and consist of the following: 1) Centration and authoritarian-based consequences; 2) Egocentric/pragmatic exchanges; 3) Interpersonal focus; 4) Societal regulation; and 5) Societal evaluation. Detailed description of coding stages and examples are provided in previous articles [1,11]. Transition stages (1.5, 2.5, etc.) were used to account for answers that provide elements of two consecutive reasoning stages. When elements of non-consecutive stages were provided, the response was coded according to the highest schema detected. The sociomoral reasoning maturity score (0 to 80 points) was obtained by summing the 16 justification scores. This test has adequate inter-rater reliability and construct validity [36]. Two trained raters scored the justifications independently. The inter-rater reliability for a proportion of the justifications (15%) was Kappa = 0.82 (*p* < .001), 95% *CI* (0.75, 0.89), which can be interpreted as an “almost perfect agreement” [64]. The sociomoral decision-making score and the sociomoral reasoning maturity score were used as the main dependent variables.

#### Cognitive and affective measures

The following measures were used to control for possible confounding variables because they have been shown to be associated with social cognition in general and sociomoral reasoning in particular [12,65–67].

The Wechsler Abbreviated Scale of Intelligence (WASI) [68] provides an estimate of the intellectual quotient based on two sub-tests of the Wechsler scales: Matrix reasoning and Vocabulary. An estimate of full scale IQ (M = 100, SD = 15) was obtained for each participant.

The Interpersonal Reactivity Index [69] is a 28 item self-report questionnaire which addresses the construct of empathy multidimensionally by providing both an affective and cognitive (theory of mind) empathy subscale [70]. Items are scored using a Likert-type scale ranging from one to five. Subscores are generated for four scales: Fantasy, Perspective taking, Empathic concern, and Personal distress. Fantasy is defined as the tendency to identify with characters from fiction work (e.g. movie, book). Perspective taking, the cognitive component of empathy, is described as the ability and tendency to adopt someone else’s perspective. Empathic concern represents the extent to which someone tends to be concerned for other’s wellbeing. Personal distress represents the emotional component of empathy and the tendency to feel discomfort or anxiety when observing someone else feeling negative emotions. The IRI factor structure is well documented [71] and the measure has good internal consistency, (alphas 0.68 to 0.79, [72,73]). Each of the IRI subscales also has good test-retest reliability with correlation coefficients ranging from .61 to .81 [72]. Furthermore, the IRI correlates with other measures of empathy, supporting its construct validity [72]. Scores for all four subscales were used in the statistical analyses: Fantasy (IRI-F), Perspective taking (IRI-PT), Empathic concern (IRI-EC), and Personal distress (IRI-PD).

The Toronto Alexithymia Scale [74] is a self-report questionnaire composed of 20 items using a Likert-type scale ranging from one to five. It measures participants’ ability to identify, understand, describe and communicate the emotions they feel. A low alexithymia score indicates a better understanding of one’s emotions. The TAS provides scores for three subscales: Difficulty Describing Feelings, Difficulty Identifying Feeling, and Externally-Oriented Thinking. Each of these factors has adequate internal consistency with alphas of .78, .75 and .66, respectively. The test-retest reliability for the full scale is .77 [75]. The French translation by Loas, Fremaux, and Marchand [76] was used in the current study (Cronbach's alpha = 0.79, correlations between items and the total score ranges from .79 (p <0.05) and .69 (p <0.007) with a mean of .52).

Given the likelihood that participants respond favourably on measures of sociomoral decision-making to please the examiner [77], participants also completed the Social Desirability Scale—17 [78]. It is composed of 17 dichotomous questions (true or false) measuring the extent to which participants tend to present socially positive images of themselves. The scale is similar to Crowne and Marlowe’s social desirability scale [79], but includes more contemporary content [80]. A total social desirability score was assigned to each participant. The questionnaire has adequate convergent validity, correlating between .52 and .85 with other commonly used measures of social desirability (Eysenck Personality Questionnaire-Lie Scale, Marlowe-Crowne Scale) [78,80].

#### Eye tracking

Eye movements and pupil dilation were recorded using a Tobii T60XL eye-tracker during the Somoral task. The eye-tracker has a sampling rate of 60 Hz, an accuracy of 0.5 degrees, and a spatial resolution of 0.35 degrees. The stimuli were presented on the custom Tobii 24-inch screen with a resolution of 1920 x 1080 pixels. Participants were positioned at a distance of 60 cm from the screen. The use of immobilisation such as a chin rest was not required, but participants were asked to limit their head movements as much as possible during the experiment.

As primary social cues [51–53,81], the faces of the characters included in the sociomoral dilemmas were defined as regions of interest (ROI). For each picture used in the experiment, ROIs were delimited manually using Tobii’s eye tracking software tool (Tobii Studio 3.2). The size of each ROI was measured in pixels so it can be used in statistical analyses.

Two metrics were extracted from the gaze data: *fixations before* (FB) and *fixation count* (FC). The metrics were extracted using the Tobii Studio software. The fixations were identified using the Velocity-Threshold Identification (I-VT) fixation classification algorithm [82]. FB represents the amount of fixations produced between the onset of each picture and the first fixation on a ROI. In the case where several faces were present in a picture, the shortest FB for each picture was used. FB was calculated for the three pictures presented in each sociomoral dilemma and then averaged to obtain one score for each dilemma. This measure is generally associated with the ability of a stimulus to stand out relative to its environment and to capture the attention of a person [83]. It would therefore be associated with bottom-up, stimuli-driven, effect such as pre-attentional capture. FC represents the total number of fixations produced within a ROI. When a picture contained several faces, FC on every face was summed to obtain a total score. FC for the three pictures were then added to obtain one score for each dilemma. FC has been associated with difficulty in extracting information from the stimulus and with the presence of more engaging information [83].

Pupil diameter was also measured continuously during every dilemma. For each participant, a segment including the presentation of the three pictures was extracted for every dilemma. Raw data were then processed according to the procedure developed by Jackson and Sirois [84]. A digital low-pass filter with a sample frequency to cut frequency ratio of 12.5 was initially applied to the raw data in order to reduce noise and variability inherent to this type of measurement. The filter was applied twice (once forward and once backward) to ensure that processing did not cause any phase shift in the signal. Missing data (attributable to eye blink, measurement error, or to the participant looking away from the screen) were then interpolated. As pupil diameter from both eyes is highly correlated [84], when samples from a single eye were missing, the samples from the other eye were used for the interpolation. Linear interpolation was then conducted using the average value of the three samples preceding and following the break. The data for the left eye and for the right eye were then combined to obtain a single average signal on which the analyses were conducted. Overall, this procedure accounts for missing data and reduces noise, while preserving the dynamic properties of the signal for each trial. For each segment, the average pupillary diameters for the complete stimuli presentation were calculated.

#### Procedure

Participants were recruited at academic institutions (colleges and universities) and testing took place in a quiet designated testing room. The entire experiment took place in a single two-hour session. Participants first completed the self-report questionnaires and other psychometric measures (WASI, IRI, TAS, SDS-17). The Tobii eye-tracker nine points calibration procedure was then performed for each participant. Calibration was verified for each point and was repeated if a deviation of more than one degree of visual angle was observed between gaze direction and measurement. Participants then completed the SoMoral task while their eye movements were measured.

#### Statistical analyses

All statistical analyses were conducted using IBM SPSS Statistics 21. The experimental design involved repeated observations (i.e. multiple dilemmas), thus mixed models were adjusted to determine the effect of eye movements on sociomoral reasoning (mixed binary logistic regression for sociomoral decision-making and mixed linear regression for sociomoral maturity) while accounting for the random effect of participants. Mixed models are appropriate in this context because they allow interpretable tests of different factors (eye movement, control variables) despite multiple observations and the non-independence of the sociomoral decision-making and sociomoral maturity scores for a single participant.

Before carrying out the main statistical analyses, control analyses were conducted to ensure that the results obtained are interpretable and attributed to the variables of interest (visual encoding) and not explained by dispositional or demographic confounding variables. Binary logistic regressions were conducted using the generalized linear mixed model procedure in SPSS (GENLINMIX) to detect possible predictors of sociomoral decision-making. Participants’ age, gender, IQ, social desirability (SDS-17), empathy (IRI) and alexithymia (TAS) were individually tested as independent predictors with decision-making (adaptive vs non-adaptive) as the dependant outcome.

The same precautions were taken for sociomoral maturity. Because it is a continuous score, mixed linear regression were conducted using the Linear mixed model procedure in SPSS (MIXED). Participants’ age, IQ, social desirability (SDS-17), empathy (IRI) and alexithymia (TAS) were individually tested as independent variables with sociomoral maturity level as the dependant outcome. Scores that were significant predictors of any aspect of sociomoral reasoning (decision-making, maturity) were added as covariates in the model for the main analyses. To ensure that the quantified relationship between eye movements and sociomoral reasoning were attributable to volitional visual encoding and not to low level stimuli-driven processes, the size of the social cues (ROI) contained in the stimuli, in pixels, was added as a covariate as well.

For the main analyses, a first set of analyses was conducted to assess the link between eye movements and sociomoral decision-making. Binary logistic regressions were conducted using the generalized linear mixed model procedure in SPSS (GENLINMIX). Specifically, eye movement variables (FB and FC) and pupillary dilation were used as independent predictors and sociomoral decision-making score (adaptive = 1, non-adaptative = 0) was used as the dependent outcome. Dilemmas were defined as the repeated measure and Participant as the random factor. The VIF for the independent variables (FB, FC, pupil dilation and ROI size) were all <2 and the condition index was <30, so the predictors were all included in the same model.

The link between the encoding of visual information and sociomoral maturity was addressed by conducting linear mixed regressions using the Linear mixed model procedure in SPSS (MIXED). Eye movements (FB, FC) and pupillary response were entered as independent variables (fixed factors) and sociomoral maturity level as a dependent outcome. Dilemmas were defined as the repeated measure. In accordance with our theoretical hypothesis (that visual encoding of social cues is linked with sociomoral maturity level independently of the dilemma), a random intercept, fixed-slope, model was used. Assuming the random effects are independent, a variance components covariance structure was used. The independent variables (FB, FC, pupil dilation and ROI size) were all included in the same model.

## Results

Participants obtained a mean sociomoral decision-making score of 14.31 (SD = 1.80) and thus produced on average 89.44% socially adaptive decisions. Their average sociomoral maturity score was 44.75 (SD = 9.45), and the corresponding average sociomoral maturity stage was therefore 2.80 (SD = 1.25). Eye-tracking data were cleaned and 143 trials were dismissed (14.90%) because no FB was available. The proportion of lost data for the included trials was 16.84% (SD = 21.17), which is not uncommon for this model of eye-tracker [85]. The average FB was 2.40 (SD = 0.91) and the average FC was 6.42 (SD = 2.59). The average Pupil dilation was 3.92 millimetres (SD = 0.73).

### Control of confounding variables

Tables 1 and 2 present the results of the predictions of the main variables of interest by possible confounding variables. None of the demographic and control variables (age, IQ, SDS-17, IRI, TAS scores) were found to be significant predictors of sociomoral decision-making or sociomoral maturity. ROI size was found to be a marginal predictor of sociomoral decision-making (*p* = .052) and was thus included as a covariate for the main analyses.

**Table 1:** Prediction of sociomoral decision-making by control variables using binary logistic regressions.

	*b (SE)*	*F*	*p*
**Age**	0.02 (0.03)	0.69	.405
**ROI size**	0.06 (0.03)	3.79	.052
**IQ**	0.01 (0.02)	0.22	.640
**Gender**	-0.25 (0.32)	0.62	.432
**SDS-17**	0.03 (0.05)	0.24	.622
**IRI—Total**	0.02 (0.01)	2.29	.130
**IRI-PT**	0.02 (0.03)	0.63	.428
**IRI-PD**	0.03 (0.03)	0.73	.394
**IRI-FS**	0.03 (0.02)	1.26	.262
**IRI-EC**	0.05 (0.03)	3.11	.078
**TAS**	-0.02 (0.02)	1.03	.310

Note: Each variable is tested individually for this step. IQ = Intellectual Quotient, SDS-17 = Social Desirability Scale– 17, IRI = Interpersonal Reactivity Index, PT = perspective taking, PD = personal distress, FS = fantasy scale, EC = empathic concern, TAS = Toronto Alexithymia Scale, SE = standard error.

**Table 2:** Prediction of sociomoral maturity by control variables using linear mixed regressions.

	*b (SE)*	*F*	*p*
**Age**	0.01 (0.01)	0.14	.713
**ROI size**	0.01 (0.01)	1.62	.203
**IQ**	0.01 (0.01)	1.88	.177
**Gender**	-0.08 (0.15)	0.26	.613
**SDS-17**	-0.01 (0.03)	0.10	.751
**IRI—Total**	0.00 (0.01)	0.06	.802
**IRI-PT**	0.01 (0.01)	0.16	.691
**IRI-PD**	0.00 (0.01)	1.28	.977
**IRI-FS**	-0.00 (0.01)	0.16	.692
**IRI-EC**	0.02 (0.01)	1.28	.262
**TAS**	-0.01 (0.01)	3.53	.070

Note: Each variable is tested individually for this step. IQ = Intellectual Quotient, SDS-17 = Social Desirability Scale– 17, IRI = Interpersonal Reactivity Index, PT = perspective taking, PD = personal distress, FS = fantasy scale, EC = empathic concern, TAS = Toronto Alexithymia Scale, SE = standard error.

### Visual encoding and sociomoral decision-making

Eye movement (FB, FC) and pupil dilation were tested as predictors of sociomoral decision-making (Table 3). FC was a significant predictor of sociomoral decision-making, OR = 1.07 95% *CI* (1.01, 1.13), *p* = .021. However, this effect only approached significance when ROI size was added to the model (Table 3).

**Table 3:** Prediction of moral decision-making by FB, FC, and pupil dilation when controlling for ROI size using binary logistic regressions.

	*b (SE)*	Lower	Odds Ratio	Upper	*p*
**Intercept**	1.33 (0.89)	0.65	3.77	21.85	.138
**FB**	-0.07 (0.06)	0.83	0.93	1.04	.226
**FC**	0.06 (0.03)	1.00	1.06	1.13	.071
**Pupil dilation**	0.14 (0.21)	0.77	1.15	1.74	.494
**ROI size**	0.01 (0.04)	0.94	1.01	1.10	.746

Note: Random effect covariance: *b*(SE) ; 0.56 (0.25) 95% CI = 0.24; 1.33, *p* = .023, FB = fixations before, FC = fixation count, SE = standard error.

### Visual encoding and sociomoral maturity

Eye movements and pupil dilation were tested as predictors of sociomoral maturity (Table 4). The association between *FB* and sociomoral maturity was not significant, nor was the association with pupil dilation. However, *FC* was found to be a significant positive predictor of sociomoral maturity (Table 4).

**Table 4:** Prediction of moral maturity by FB, FC and pupil dilation when controlling for ROI size using linear mixed regressions.

	*b (SE)*	95% CI	*p*
**Intercept**	3,12* (0.46)	2.21; 4.02	< .001
**FB**	-0.00 (0.02)	-0.05; 0.04	.974
**FC**	0.04* (0.01)	0.02; 0.06	< .001
**Pupil dilation**	-0.11 (0.11)	-0.33; 0.10	.299
**ROI size**	-0.02 (0.01)	-0.05; 0.00	.096

Note: CS covariance: *b*(SE) = 1.23 (0.06), *95% CI* = 1.11; 1.36, *p* < .001, *Pseudo-R2* for the model = .01

*p < .001, FB = fixations before, FC = fixation count, SE = standard error.

## Discussion

The objective of this study was to assess the relation between visual encoding of social information and sociomoral reasoning using a visual and ecological paradigm. Our main results confirm the existence, and provide a better understanding, of this relation. More specifically, this study suggests that visual encoding, as a first step in the processing of social information, is associated with a subsequent stage of sociomoral reasoning (production of a mature justification). Visual encoding predicted sociomoral maturity: greater *fixation count* on faces present in the dilemmas was associated with the production of more mature sociomoral justifications. However, prediction of sociomoral decision-making by visual encoding did not reach statistical significance when controlling for ROI size. These results do not appear to stem from either the socio-demographic or dispositional characteristics of participants, nor are they accounted for by other socio-cognitive aspects such as empathy or theory or mind (imputation of mental state to oneself or to others [86]), or by the situational characteristics of the stimuli such as their size. In summary, eye movements appear to be reliable proxies for the cognitive processes playing a significant role in sociomoral reasoning, and especially sociomoral maturity.

### Links with previous research on moral reasoning

A significant association was established between sociomoral maturity and visual encoding. To our knowledge, no other study has addressed the link between visual encoding and sociomoral maturity. Studies evaluating the nature and quality of the justification produced using Neo-Kohlbergian tests such as the Defining Issues Test 2 (DIT-2), for example, have not focused on the role of visual encoding, nor on the role of social information processing and have not used paradigms that allow this. Conversely, more recent sociomoral reasoning studies that use eye tracking to address sociomoral decision-making do not include an objective measurement of the justification produced by the participants. The current findings suggest that the cognitive process leading to the production of a relevant and socially convincing justification likely begins at the first stages of information processing. The establishment of similar patterns of association between visual encoding and moral processes in studies focusing on the production of a justification (e.g. SoMoral) and those focusing on decision-making (e.g., Trolley Problem) suggests a link between intuitive and deliberative aspects of moral reasoning. This challenges the idea that justifications of moral decision are only produced *a posteriori* and are completely independent of moral decision-making.

The association between visual encoding and the production of socially adaptive decisions was not significant when controlling for ROI size. These results contrast with studies in social cognition that have found a link between visual encoding of social cues and social abilities [46,47,49], as well as those in children and in clinical populations (e.g., autism spectrum disorders, opposition disorder) using self-report or observer report of behavior and social adaptation [50,87]. The current findings also differ from those of studies using trolley-type dilemmas that report differential fixation durations depending on the chosen moral option [58–61]. Notably, the outcome measures used across these studies are not equivalent and could account for these differences. Past studies compared deontological responses to utilitarian ones, whereas the SoMoral compares socially adaptive and non-adaptive responses.

It was expected that pupillary dilation would be related to sociomoral reasoning. However, no relation–with either decision-making or sociomoral maturity—reached statistical significance. This result is unexpected considering that pupillary dilation is known to be a reliable measure of physiological arousal and that physiological arousal is associated with sociomoral reasoning [22–24]. One possible explanation is that the intensity of the sociomoral issues presented in the sociomoral dilemmas is lesser than that of classical, extreme dilemmas such as the trolley problem. Dilemmas describing life or death situations are likely to induce stronger emotions and to elicit heightened emotional appraisal compared to dilemmas representative of everyday life, which may be associated with a significant increase in sympathetic activity. Alternatively, some studies suggest that it is not physiological arousal that predicts sociomoral reasoning as such, but its perceived intensity by the participant. For example, studies using false biofeedback indicate that an increase in perceived heart rate is associated with more frequent production of sociomoral action [22,88]. Thus, the intensity of the physiological arousal induced by the dilemmas in the SoMoral task is possibly not important enough to be processed by the participants as relevant information for sociomoral reasoning.

This study emphasizes the relevance of using an ecological paradigm to address the issue of visual encoding in sociomoral reasoning. The use of dilemmas representative of everyday life provides an opportunity to gather more realistic social information from participants, and this may account for some of the differences in findings between previous research on sociomoral reasoning and the current study. As such, using the SoMoral task addresses some of the issues related to the improbability of paradigms such as the trolley problem and contributes to the advent of sociomoral psychology that is less hypothetical and more applicable to everyday life.

### Theoretical implications of the results

The study highlights interesting distinctions between predictors of sociomoral decision-making versus predictors of sociomoral maturity. Our results suggest that sociomoral maturity is specifically associated with volitional visual encoding strategies, but not with low-level perceptive processes such as pre-attentional capture. It offers some support for dual-process models of sociomoral reasoning and the idea that, while sociomoral decision-making may be based on intuition, the production of a justification relies on higher-level processes. Although the distinction between the different aspects of sociomoral reasoning is not complete, the specific association of fixation count and sociomoral maturity is consistent with the idea that sociomoral decision-making and sociomoral maturity, although sharing some common features, are two distinct constructs [89–91]. Using the SoMoral task in the current study possibly highlights this distinction since participants were first asked about their presumed actions (“What would you do?”), and then were asked to justify (“Why?”), two distinct questions that may involve different cognitive processes. This is consistent with studies reporting that people tend to produce deontological judgments when asked to choose a morally acceptable option (moral judgement) and to make utilitarian decisions when faced with the situation (moral decision-making) [91,92]. This approach is novel because it allows measurement of both questions for each dilemma.

### Limitations of the study

This study presents some limitations that must be taken into account when interpreting and generalizing its results. First, although the stimuli are visual, the formulation of a justification relies on verbal expression abilities to some extent. A previous study using the same task found that level of sociomoral maturity correlates with verbal fluency [11]. It is therefore probable that an individual with better verbal fluency may be able to provide justifications of a higher level of maturity. Also, the fact that most participants were recruited in academic institutions introduces a bias since verbal skills and verbal fluency are associated with education [93]. Since our statistical analyses address each response individually and not as an average score for each participant, and since there is no way to ask for a justification without using language, we can assume that the influence of this variable is optimally kept to a minimum. Second, the use of a non-experimental design limits the scope of the results obtained. The relation between visual encoding and sociomoral reasoning remains statistical and therefore the nature and significance of the contribution of visual encoding is not clear. A possibility remains that people who tend to generate more socially adaptive responses and to provide more mature justifications are also people who tend to pay more attention to social cues.

## Conclusion

This study suggests that visual encoding of social cues is a key step in sociomoral reasoning. The use of ecological dilemmas with eye tracking is innovative and especially useful for the generalization and interpretation of results in sociomoral psychology. It also demonstrates the relevance of studying both sociomoral decision-making and sociomoral maturity in the context of an integrative social cognitive understanding of sociomoral reasoning. Questions remain about the exact nature of the association between visual encoding and sociomoral reasoning. In particular, the use of an experimental design could confirm the presence of a cause-and-effect mechanism linking visual encoding and sociomoral reasoning. Nevertheless, this study constitutes a step forward the establishment of connexions between the domains of perception, sociomoral reasoning, social cognition and decision-making.

## Supporting information

S1 Dataset DataPlosOne. (XLSX)
